# Contrast-Free Myocardial Infarction Segmentation with Attention U-Net

**DOI:** 10.3390/diagnostics16050768

**Published:** 2026-03-04

**Authors:** Khaled Ali Deeb, Yasmeen Alshelle, Hala Hammoud, Andrey Briko, Vladislava Kapravchuk, Alexey Tikhomirov, Amaliya Latypova, Ahmad Hammoud

**Affiliations:** 1 Department of Information Processing and Management Systems, Bauman Moscow State Technical University, 105005 Moscow, Russia; 2 Department of Medical and Technical Information Technology, Bauman Moscow State Technical University, 105005 Moscow, Russia; 3 Department of Biomedical Technologies, Bauman Moscow State Technical University, 105005 Moscow, Russia

**Keywords:** cardiac MRI, segmentation, deep learning, myocardial infarction, CNN, U-Net attention, cine CMR

## Abstract

**Background:** Cardiovascular magnetic resonance (CMR) is the clinical gold standard for assessing cardiac anatomy and function. However, the manual segmentation of cardiac structures and myocardial infarction (MI) is time-consuming, prone to inter-observer variability, and often depends on contrast-enhanced imaging. Although deep learning (DL) has enabled substantial automation, challenges remain in generalizability, particularly for MI detection from non-contrast cine CMR. **Objective:** This study proposes a comprehensive DL-based framework for automatic segmentation of cardiac structures and myocardial infarction using contrast-free cine CMR. **Methods:** The framework integrates multiple convolutional neural network (CNN) architectures for cardiac structure segmentation with an attention-based deep learning model for MI localization. Post-processing refinement using stacked autoencoders and active contour modeling is applied to improve anatomical consistency. Segmentation performance is evaluated using overlap-based and boundary-based metrics, including the Dice Similarity Coefficient (DSC), Mean Contour Distance (MCD), and Hausdorff Distance (HD). **Results:** The best-performing model achieved Dice scores of 0.93 ± 0.05 for the left ventricular (LV) cavity, 0.89 ± 0.04 for the LV myocardium, and 0.91 ± 0.06 for the right ventricular (RV) cavity, with consistently low boundary errors across all structures. Myocardial infarction segmentation achieved a Dice score of 0.80 ± 0.02 with high recall, demonstrating reliable infarct localization without the use of contrast agents. **Conclusions:** By enabling accurate cardiac structure and myocardial infarction segmentation from contrast-free cine CMR, the proposed framework supports broader clinical applicability, particularly for patients with contraindications to gadolinium-based contrast agents and in emergency or resource-limited settings. This approach facilitates scalable, contrast-independent cardiac assessment.

## 1. Introduction

Cardiovascular diseases (CVDs) remain the leading cause of mortality worldwide, accounting for approximately 17.9 million deaths annually, corresponding to nearly 31% of global mortality [1]. Coronary artery disease and stroke represent the dominant contributors to this burden [2,3]. Accurate and timely diagnosis is therefore essential for improving patient outcomes, and medical imaging plays a central role in disease detection, characterization, and longitudinal assessment [4]. Among available modalities, cardiovascular magnetic resonance (CMR) is widely regarded as the reference standard for non-invasive evaluation of cardiac anatomy, function, and tissue characteristics due to its high spatial resolution, excellent soft-tissue contrast, and the absence of ionizing radiation [5,6,7]. Quantitative assessment of cardiac function—such as left ventricular ejection fraction (LVEF), ventricular volumes, and myocardial mass—relies on precise segmentation of the left ventricle (LV), right ventricle (RV), and myocardium [8]. In clinical practice, these structures are commonly delineated manually, a process that is time-consuming and prone to inter- and intra-observer variability, particularly in anatomically challenging basal and apical slices where boundaries are often poorly defined [9,10].

To address these challenges, cardiac image analysis has progressively transitioned from traditional atlas-based and deformable model approaches [11,12,13] to deep learning (DL), particularly convolutional neural networks (CNNs) [14,15,16,17,18]. Architectures such as U-Net and its extensions—including recurrent, 3D, and attention-based variants—have demonstrated strong performance for cardiac structure segmentation in cine and late gadolinium enhancement (LGE) MRI [19,20,21,22,23]. Large-scale studies illustrate the maturity of these methods: Bai et al. [10] reported near-expert-level segmentation accuracy on UK Biobank cine CMR using fully convolutional networks, while Davies et al. [24] demonstrated robust, multi-institutional performance across diverse scanners and patient cohorts. Nevertheless, important limitations persist. Many models exhibit degraded performance in regions with high anatomical variability, particularly near the cardiac base and apex [25], and encoder–decoder feature misalignment can lead to imprecise boundary delineation in multi-structure segmentation tasks [26]. Furthermore, generalizability across heterogeneous populations, imaging protocols, and disease phenotypes remains an open challenge [27].

A particularly difficult and clinically relevant problem is myocardial infarction (MI) assessment using contrast-free cine CMR. While LGE imaging remains the clinical standard for infarct visualization, contrast administration may be contraindicated, unavailable, or impractical in certain settings, including patients with renal impairment or in time-critical or resource-limited workflows. In cine CMR, infarcted myocardium does not exhibit explicit intensity enhancement; instead, pathological regions manifest as subtle alterations in myocardial motion and signal characteristics, making direct infarct localization considerably more challenging than in contrast-enhanced imaging [28].

Earlier MI segmentation studies have primarily focused on LGE or multi-sequence data [29,30], limiting their applicability to contrast-free cine MRI.

Recent work has explored contrast-free infarct assessment through cine-to-LGE or cine-to-enhancement generative frameworks. Qi et al. [31] introduced cine-generated enhancement (CGE) to synthesize LGE-equivalent images from cine CMR, while Qi et al. [32] proposed a diffusion-based approach for generating synthetic enhancement images to support infarct quantification. Similarly, Demirel et al. [33] and Zhang et al. [34] demonstrated the feasibility of virtual native enhancement (VNE) and generative AI methods for scar visualization without contrast administration. These approaches represent an important advance toward contrast-free infarct assessment; however, their primary objective is image synthesis or enhancement, whereas the present study directly optimizes segmentation robustness, boundary accuracy, and anatomical consistency on native cine CMR without generating synthetic contrast images. In particular, generative methods do not explicitly evaluate how segmentation robustness, boundary accuracy, and anatomical plausibility are affected by architectural design choices when operating directly on cine data, nor do they enforce anatomically consistent contours in challenging regions such as basal and apical slices.

In parallel, the availability of annotated myocardial infarction labels in cine CMR remains inherently limited, as infarct annotation typically relies on contrast-enhanced reference sequences and expert interpretation [35]. Consequently, publicly available cine-based MI datasets are small and highly imbalanced, motivating methodological approaches that remain robust under realistic, limited-data conditions rather than relying on large-scale infarct-positive cohorts.

In this work, we propose a unified deep learning framework for contrast-free cine CMR that addresses both cardiac structure segmentation and myocardial infarction localization. Rather than introducing new convolutional building blocks, the novelty of this study lies in a systematic and controlled benchmarking of CNN architectural depth, filter capacity, and pooling strategy for multi-structure cardiac segmentation under identical training conditions, combined with stacked autoencoder-based contour refinement to enhance anatomical consistency. In addition, we perform direct infarct localization from native cine CMR using an attention U-Net augmented with squeeze-and-excitation (SE) modules, avoiding reliance on synthetic contrast or image generation.

The main contributions of this study are:

(1) Controlled CNN benchmarking for cardiac structure segmentation, evaluating shallow, deeper, larger, and max-pooling architectures for LV cavity, LV myocardium, and RV cavity segmentation, with emphasis on anatomically challenging basal and apical regions.
(2) Direct contrast-free myocardial infarction localization, using an attention U-Net with SE blocks to enhance spatial and channel-wise sensitivity to subtle cine-derived infarct cues.
(3) Post-processing refinement for anatomical plausibility, introducing a three-stage stacked autoencoder (SAE) pipeline followed by active contour modeling to correct topological inconsistencies and improve boundary smoothness.

Overall, the proposed framework provides a contrast-independent solution for integrated anatomical and pathological cardiac segmentation from routine cine CMR. By focusing on segmentation robustness, boundary accuracy, and limited-data feasibility, this work complements recent generative enhancement approaches and supports broader clinical applicability where contrast-enhanced imaging may be unavailable or contraindicated.

## 2. Materials and Methods

### 2.1. Data Source and Ethical Compliance

This study utilized two publicly available cardiac cine MRI datasets: the Sunnybrook Cardiac Dataset (SCD) [36] and the Kaggle Second Annual Data Science Bowl dataset [37]. Both datasets consist of fully anonymized scans from healthy individuals and patients diagnosed with various cardiovascular diseases (CVDs), including MI. A cardiologist manually annotated detailed endocardial and epicardial borders for both the diastolic and systolic phases. These annotations were subsequently validated by a radiologist to ensure anatomical accuracy and reliability for machine learning applications [38]. Formal inter-observer variability analysis was not performed, as only a single set of expert annotations was available for each dataset. However, independent validation was used to minimize annotation inconsistencies and ensure anatomical plausibility.

The datasets comply with the ethical standards outlined in the Declaration of Helsinki, and no additional institutional approval was required for secondary analysis. A total of 880 annotated image-label pairs were used for training and evaluation, as shown in Table 1. The dataset was split into three subsets: training (70%), validation (15%), and test (15%). The training set was used for model training, while the validation set was employed for hyperparameter tuning and model selection to ensure that the model’s performance could be evaluated on unseen data. The test set, which was not used during training or validation, was reserved for the final model evaluation to assess generalizability. This split ensures a robust evaluation of the model’s performance on unseen data and minimizes the risk of overfitting.

While the datasets used in this study are public and curated, it is important to acknowledge certain limitations regarding their generalizability. Public datasets, such as SCD and the Kaggle Data Science Bowl dataset, are often curated with controlled imaging protocols and standardized scanning environments. As a result, they may not fully represent the variability encountered in real-world clinical settings. Clinical imaging data can exhibit variability due to differences in scanner models, vendors, acquisition parameters, and other operational factors, which can impact factors like image quality, resolution, and contrast. This variability could potentially affect the model’s ability to generalize to new, unseen clinical cases. Therefore, while the results obtained from these datasets are promising, additional validation with datasets derived from multiple scanner types and clinical settings would provide a more robust understanding of the model’s generalizability and clinical applicability. Future work could benefit from incorporating such multi-vendor clinical data to further test the model’s performance across a broader range of clinical conditions.

In addition to ethical compliance, the clinical deployment of contrast-free myocardial infarction (MI) detection tools requires careful consideration of regulatory readiness. Although this study demonstrates the feasibility of using cine CMR for MI localization, regulatory approval from relevant health authorities (such as the FDA or CE) will be required before clinical use. This process involves extensive validation in clinical settings, multi-center studies, and testing across multiple scanner types and vendors. Moreover, regulatory requirements for medical devices, including compliance with medical device standards, data privacy laws, and ensuring system safety and efficacy, must also be met before clinical deployment.

The training process and model evaluation methodology have been designed to minimize overfitting. Specifically, the study incorporated several regularization techniques due to the limited number of infarct-positive slices (75 images). These regularization strategies included data augmentation, such as random rotations, horizontal flips, and spatial shifts, to artificially increase the diversity of the training set. Additionally, early stopping was implemented to monitor validation performance, halting training when no further improvements were observed. To further ensure robust generalization, L2 regularization was applied to prevent the model from overfitting to noise or specific artifacts present in the data.

In addition to ethical compliance, the clinical deployment of contrast-free myocardial infarction (MI) detection tools requires consideration of regulatory readiness. While this study demonstrates the feasibility of using cine CMR for MI localization, it is important to acknowledge that regulatory approval from relevant health authorities (such as the FDA or CE) will be required for clinical use. This process typically involves extensive clinical validation, including multi-center and multi-vendor studies, to ensure that the system performs reliably across different scanners and clinical environments. Regulatory considerations will also include compliance with medical device standards and data privacy laws.

This study acknowledges that the number of infarct-positive slices is limited, and while this limitation reflects the scarcity of expert-annotated MI regions in cine CMR datasets, it mirrors the imbalanced data often encountered in real-world clinical applications. The results of this study, therefore, emphasize robustness under limited-data conditions, reflecting the constraints that typically exist in clinical environments rather than relying on large-scale, highly curated supervised learning datasets.

**Figure 1:**
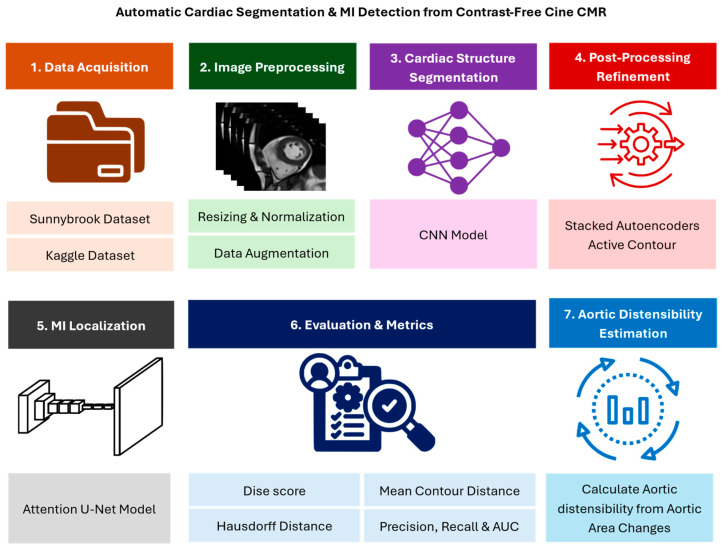
Overview of the proposed pipeline for automatic cardiac segmentation and myocardial infarction detection from contrast-free cine CMR. The framework integrates data acquisition, image preprocessing, CNN-based cardiac structure segmentation, post-processing refinement using stacked autoencoders and active contours, attention-based myocardial infarction localization, quantitative evaluation, and downstream aortic distensibility estimation. The schematic illustrates the modular and sequential nature of the pipeline used throughout this study.

To provide a comprehensive understanding of the methodology, Figure 1 illustrates the complete end-to-end pipeline for automatic cardiac structure segmentation and myocardial infarction detection from contrast-free cine CMR. This pipeline encompasses data acquisition, preprocessing, CNN-based segmentation of cardiac structures (LV cavity, LV myocardium, and RV cavity), post-processing refinement using stacked autoencoders and active contour modeling, and myocardial infarction localization using an attention-based U-Net model. Outputs from the model are evaluated using both overlap-based and boundary-based metrics, and the refined segmentation masks are subsequently used for downstream functional analysis, such as aortic distensibility estimation.

As part of the complete pipeline, downstream functional analysis, such as aortic distensibility estimation, was performed using the segmentation results of the ascending and descending aorta. This analysis is intended as a validation of segmentation utility, demonstrating that accurate cardiac structure segmentation enables reliable and clinically relevant downstream measurements.

All segmentation tasks in this study are learned and evaluated directly from native cine CMR. The framework emphasizes direct segmentation rather than contrast enhancement or image synthesis, enabling controlled analysis of CNN architectural design choices and post-processing strategies on segmentation robustness and boundary accuracy. By avoiding intermediate enhancement representations, the approach preserves a direct correspondence between cine image characteristics and anatomical predictions.

### 2.2. Image Preprocessing

All cine MRI frames were resampled to 256 × 256 pixels and intensity-normalized to ensure consistency across scans. Ground truth annotations were converted into binary masks and used during training and evaluation. These masks were employed to define regions of interest (ROIs) centered on the relevant cardiac structures for supervised learning. During inference, ROIs were generated exclusively from model-predicted segmentation outputs, and no ground truth information was used at any stage of testing or deployment. Each ROI was resized to 64 × 64 pixels for CNN input, with corresponding mask representations reshaped to 32 × 32 pixels and subsequently refined at higher spatial resolution during post-processing.

The preprocessing pipeline therefore comprises two sequential resampling stages. First, full cine MRI frames are standardized to 256 × 256 pixels to harmonize spatial resolution across subjects and datasets and to enable consistent ROI extraction. Second, following ROI localization, the extracted regions are resized to the network input resolution. These stages serve distinct purposes—dataset harmonization and computationally efficient localized learning—and are applied sequentially rather than redundantly.

Morphological filtering was applied to suppress fragmented or spurious segmentations and improve mask quality prior to training [39]. Specifically, binary morphological opening followed by closing was performed using a disk-shaped structuring element with a radius of 2 pixels, removing small isolated artifacts and smoothing region boundaries. No contrast enhancement was applied at any stage, ensuring compatibility with clinical workflows that avoid gadolinium administration.

Interpolation was performed in a data-consistent manner to minimize resampling artifacts and prevent boundary distortion. Bilinear interpolation was used for grayscale cine MRI intensity images to preserve continuous intensity transitions, while nearest-neighbor interpolation was applied to binary segmentation masks to maintain label integrity and prevent class mixing. Intensity normalization was applied after full-frame resampling to reduce scanner-dependent variability, and subsequent morphological filtering further suppressed isolated artifacts.

Given the two-stage resizing process (256 × 256 → ROI → network input), particular attention was paid to preserving boundary fidelity. Although ROI-level resizing introduces spatial resampling, its potential smoothing effect is confined to the coarse segmentation stage. The final anatomical precision is restored through a multi-stage refinement strategy operating at higher spatial resolution, including stacked autoencoder-based shape regularization and active contour modeling. Importantly, segmentation accuracy was quantitatively assessed using boundary-sensitive metrics, including the Hausdorff Distance (HD) and Mean Contour Distance (MCD), as reported in Section 3.2.6. The low HD and MCD values observed for the final post-processed outputs confirm that the resampling strategy does not introduce systematic boundary bias and that contour fidelity is effectively preserved. Furthermore, the multi-stage post-processing pipeline minimizes potential distortion caused by the initial resizing stages, ensuring that boundaries are accurately refined even when small-scale changes are introduced during the preprocessing phase.

To mitigate overfitting—particularly given the limited number of MI slices—data augmentation was applied during training, including random rotations, horizontal flips, and spatial shifts. These augmentations increased the effective diversity of the training data and improved generalization.

The dataset was divided into training (70%), validation (15%), and test (15%) subsets. The training set was used for model development, the validation set for hyperparameter tuning, and the test set was reserved for final performance evaluation. The validation set, comprising 75 infarct-positive slices, was used specifically for MI localization and model optimization. This data split ensures robust evaluation under realistic clinical conditions while minimizing the risk of overfitting.

Downstream validation applications, such as aortic distensibility estimation, were performed using the segmentation results of the ascending and descending aorta. This further supports the utility of the segmentation model in clinical settings by demonstrating that accurate segmentation can be used to generate clinically relevant measurements.

### 2.3. CNN-Based Cardiac Structure Segmentation

We implemented and compared four CNN architectures to evaluate segmentation performance for key cardiac anatomical structures, including the LV cavity, LV myocardium, and RV cavity. These architectures—Shallow CNN, Max-Pooling CNN, Larger CNN, and Deeper CNN—were designed to systematically investigate the influence of filter capacity, network depth, and pooling strategy on binary mask regression performance in cine cardiovascular CMR. Each network accepts grayscale region-of-interest (ROI) input images of size 64 × 64 pixels and produces a binary segmentation mask, which is subsequently reshaped to 32 × 32 pixels. While the architectures differ in convolutional depth, number of filters, and pooling operations, they share a consistent output formulation and training protocol to ensure fair and controlled comparison.

All architectures terminate with a fully connected dense layer comprising 1024 units with sigmoid activation. Unlike fully convolutional networks that perform direct pixel-wise prediction, the proposed CNNs formulate segmentation as a mask-level regression task at the ROI scale. This design enables the dense layer to encode global anatomical shape and contextual information, which is particularly important in cine CMR, where myocardial boundaries are often weakly defined and local intensity cues alone are insufficient.

Only these four architectures are treated as distinct CNN models and are summarized in Table 2. This ReLU configuration represents a parameter-level variant of an existing architecture, introduced solely to examine optimization behavior under an alternative activation function. It is therefore not considered a separate architectural model and is intentionally excluded from Table 2.

To mitigate overfitting, L2 regularization (λ = 0.0001) was applied to the dense layer in all architectures. For transparency and reproducibility, the total number of trainable parameters for each CNN model was computed directly from the implemented layer configurations and is reported in Table 2.

#### Architectural Rationale

Cine CMR frequently exhibits weak and locally ambiguous myocardial boundaries, with segmentation errors often concentrated in anatomically variable basal and apical slices [40]. In this setting, architectural choices controlling spatial down-sampling and representational capacity are expected to influence segmentation robustness and boundary continuity. Pooling operations determine the balance between feature invariance and boundary detail: max pooling can increase robustness to local intensity and motion variability, whereas average pooling may better preserve smooth anatomical contours, potentially resulting in distinct error characteristics across slices [41]. Increasing network depth expands hierarchical feature abstraction and enlarges the effective receptive field; however, under limited-data conditions, it may also increase sensitivity to optimization instability and overfitting, which can compromise boundary completeness in challenging regions [42]. By contrast, increasing filter capacity is expected to strengthen global shape and contextual encoding at the ROI level, thereby improving regional coherence and reducing mask fragmentation. The four CNN variants were therefore designed to isolate these architectural factors under identical training and evaluation conditions.

### 2.4. Model Training and Optimization

All CNN models were trained using the Adam optimizer with a learning rate of 0.001 and a batch size of 16. The mean squared error (MSE) between the predicted and ground truth masks was adopted as the primary loss function. While Dice-based and binary cross-entropy losses are commonly used in medical image segmentation, MSE was intentionally selected to frame the segmentation task as a regression problem over binary masks. This formulation encourages smoother prediction maps and facilitates more stable optimization, which is especially beneficial for cine-based cardiac segmentation where anatomical boundaries are frequently ambiguous and characterized by gradual intensity transitions. Furthermore, the use of MSE aligns with the subsequent stacked autoencoder-based post-processing stage, which operates within a reconstruction framework and benefits from continuous-valued mask representations, as opposed to binary outputs [43].

To improve generalization and mitigate overfitting—particularly due to the limited number of infarct-positive slices (75 images)—data augmentation strategies were employed during training. These included random rotations (±50°), horizontal flips, and spatial shifts (±10%). These augmentations were applied online and independently at each training iteration, thereby increasing the diversity of the training data and reducing model sensitivity to specific spatial configurations.

Training was conducted for a fixed duration of 20 epochs across all CNN variants. This decision was informed by convergence behavior observed consistently across architectures, as detailed in Section 3.2.3. Loss trajectories demonstrated monotonic stabilization within this training window, indicating that model capacity and architectural design were the primary factors governing performance, rather than extended training duration. To ensure a fair and unbiased comparison of architectural variants, all networks were trained under identical optimization schedules, augmentation settings, and stopping criteria.

All experiments were implemented in Python 3.1 using standard deep learning libraries and executed in a cloud-based environment (Google Colab runtime, October 2025 release). Model inference was conducted on individual cine CMR slices using a single forward pass without iterative optimization. Under this setup, inference required only fractions of a second per slice, enabling efficient processing of complete cine sequences. Specifically, the average inference time per slice was approximately 0.3 s, and for a full patient scan (comprising 30 slices), the total inference time was approximately 9 s. This performance was achieved on standard cloud infrastructure, and while hardware configurations were not optimized for deployment, the system was able to process data efficiently. These details are included to support reproducibility and provide an indicative estimate of computational cost.

### 2.5. Post-Processing with Stacked Autoencoders

To enhance the spatial accuracy of CNN-generated segmentations, we employed a three-stage SAE as a dedicated post-processing refinement module, applied after the CNN-based mask prediction [44]. The SAE was designed to improve the anatomical coherence and smoothness of predicted segmentation masks by learning compact, high-level representations of segmentation shapes, rather than relying on raw image features. Initially, the SAE compresses the 64 × 64 predicted segmentation mask into a 100-dimensional latent space, capturing the most salient global and local spatial characteristics required for accurate reconstruction.

The latent dimensionality (100) was selected empirically based on preliminary validation experiments, aiming to balance compression and reconstruction fidelity. Multiple latent sizes (50, 75, 100, 150, and 200) were evaluated on the validation set. Latent sizes below 75 led to excessive information loss and degraded contour continuity, whereas dimensions above 150 showed negligible improvement in reconstruction but increased model complexity and overfitting risks. A latent size of 100 provided a stable trade-off, preserving global anatomical structure and boundary coherence while maintaining compression to suppress local noise.

A secondary SAE layer further refines this latent representation, enhancing feature abstraction and suppressing spurious local irregularities. Finally, both stacked encoders are integrated into a reconstruction stage, producing a refined segmentation mask with improved contour continuity and anatomical consistency.

It is important to emphasize that the SAE does not perform independent segmentation or image-based feature learning. Instead, it functions exclusively as a post-processing mechanism that refines the outputs of the primary CNN-based segmentation models. This design allows the SAE to exploit the strong initial localization provided by the CNN, while correcting shape discontinuities, holes, and boundary fragmentation—issues that are particularly prevalent in anatomically challenging regions such as basal and apical slices in cine CMR.

Following SAE-based refinement, an active contour (snake) model [45] was applied as the final contour regularization step, explicitly enforcing smoothness and anatomical plausibility at object boundaries. The active contour framework, a classical energy-minimization method, evolves an initial contour toward object boundaries by balancing internal smoothness constraints with external forces derived from image gradients [46]. In this study, the initial contour for the active contour model was directly initialized from the SAE-refined binary mask, ensuring stable convergence and mitigating sensitivity to initialization.

The active contour energy formulation comprises internal terms that penalize excessive curvature and contour irregularity, along with external forces derived from image gradients that attract the contour toward true anatomical boundaries. This process does not introduce additional learning or parameter optimization; rather, it operates deterministically to regularize the final segmentation geometry. By combining SAE-based shape regularization with active contour boundary refinement, the post-processing pipeline corrects residual boundary noise while preserving the anatomical structure inferred by the CNN [47]. The SAE was trained using a hybrid loss function that combines pixel-wise reconstruction accuracy with a probabilistic distributional term to balance local fidelity and global shape consistency. The loss function is defined as:

(1) $$L=\frac{\alpha }{2}\times MAE(y_{true},y_{pred})+\beta \times D_{KL}(y_{true}∥y_{pred})$$

where *α* = 0.0001 and *β* = 3, with MAE representing the mean absolute error and $D_{KL}$ representing the Kullback–Leibler (KL) divergence. KL divergence is particularly appropriate for binary segmentation masks because it measures the discrepancy between predicted and true probability distributions over foreground and background regions. While MAE captures pixel-wise reconstruction errors, the KL term enforces global structural alignment between predicted and reference masks, thereby improving anatomical coherence. This hybrid formulation enables the SAE to correct both local boundary defects and global shape inconsistencies in a unified manner.

The values of *α* and *β* were selected empirically through preliminary validation experiments using a grid-search strategy. Multiple combinations were evaluated, and the selected values provided the best trade-off between boundary smoothness and shape preservation on the validation set.

Finally, to assess robustness with respect to optimization behavior in the overall framework, different weight initialization schemes (zero, random_uniform, and random_normal) were evaluated for the CNN models feeding into the SAE-based post-processing pipeline. These initialization experiments are reported separately in Section 3 and are not part of the SAE optimization itself.

### 2.6. MI Segmentation Using Attention U-Net

For infarct localization, we designed an Attention U-Net architecture enhanced with squeeze-and-excitation (SE) blocks to strengthen channel-wise and spatial attention mechanisms. The model was trained on a subset of 75 infarction-positive cine CMR slices. Inputs were cropped around the LV and myocardium using the ROI extraction strategy described in Section 2.2, resized to 128 × 128 × 3, and intensity-normalized.

Although the original cine MRI data are grayscale, the input was constructed by replicating the grayscale image across three channels to match the architectural requirements of standard convolutional backbones designed for three-channel inputs. This approach preserves intensity information while maintaining compatibility with established CNN implementations.

The encoder follows a conventional U-Net structure with dense convolutional feature extraction, augmented by SE blocks to perform adaptive channel recalibration. The decoder mirrors the encoder and incorporates attention gates within the skip connections to suppress irrelevant activations and emphasize infarct-relevant regions. A final 1 × 1 convolution layer with sigmoid activation produces the binary segmentation probability map, enabling pixel-wise infarct prediction.

During inference, ROIs are generated from predicted cardiac structure segmentations rather than ground truth masks. While this introduces a theoretical risk of error propagation from early-stage segmentation to infarct localization, several design choices mitigate this effect.

First, ROI extraction is based on bounding-box localization with fixed padding margins rather than precise contour cropping. This strategy preserves contextual myocardial information even when minor segmentation inaccuracies occur, reducing sensitivity to small boundary deviations.

Second, the Attention U-Net operates on resized ROIs that retain surrounding myocardial context, allowing the model to learn infarct-relevant motion and texture patterns beyond exact contour boundaries. Thus, the infarct segmentation network does not depend on pixel-perfect upstream segmentation.

Third, the SE blocks and spatial attention gates dynamically recalibrate feature responses within the ROI, enabling the model to suppress irrelevant background regions and emphasize informative myocardial features. This attention-driven refinement increases robustness to moderate localization errors in the initial segmentation stage.

Finally, the overall pipeline was quantitatively validated using boundary-sensitive metrics (HD and MCD) for structural segmentation (Section 3.2.6) and overlap-based metrics with confidence intervals for MI localization (Section 3.3.2), confirming that error propagation does not produce systematic degradation in final infarct segmentation performance.

SE blocks were inserted after major convolutional blocks within the encoder and bottleneck stages. This placement allows recalibration of feature maps after spatial feature extraction but before resolution reduction. Empirically, channel attention applied at intermediate feature depths has been shown to improve discriminative capacity in medical image segmentation tasks, particularly when foreground regions are small and class imbalance is pronounced [48].

By recalibrating channel-wise feature responses at progressively abstract representation levels, the network enhances infarct-relevant feature channels while suppressing redundant activations. This is especially important in contrast-free cine CMR, where infarcted regions are characterized by subtle motion abnormalities rather than strong intensity contrast.

The attention mechanism operates across both spatial and channel dimensions. Let $F\in R^{H\times W\times C}$ denote the input feature map, where H, W, and C represent the height, width, and number of channels, respectively.

The spatial attention map, $A_{\mathrm{spatial}}(F)$, is computed as:

(2) $$A_{spatial}(F)=\sigma (Conv(Concat(F_{1},F_{2}))),$$

where $F_{1}$ and $F_{2}$ are feature maps obtained by applying different convolutions to the input F, and σ represents the sigmoid activation function. This spatial attention mechanism allows the model to focus on specific regions of the feature map that are deemed more important for the segmentation task.

The channel attention map, $A_{\mathrm{channel}}(F)$, is obtained by first applying global pooling to the feature map F, followed by a fully connected layer and a sigmoid activation:

(3) $$A_{channel}(F)=\sigma (FC(GlobalPooling(F))),$$

where GlobalPooling aggregates the spatial information along each channel, and FC represents a fully connected layer that generates channel-wise attention weights [48]. Sigmoid activation ensures that the attention values lie within the range (0, 1).

The final output feature map, $F_{\mathrm{output}}$, is obtained by applying both the spatial and channel attention maps to the original feature map:

(4) $$F_{output}=A_{spatial}(F)\times A_{channel}(F)\times F,$$

This operation recalibrates the feature map by focusing on the most relevant spatial regions and channel-wise features, improving the model’s ability to localize the myocardial infarction.

A detailed, layer-by-layer architectural description can be found in Table 3.

### 2.7. Aortic Distensibility Estimation

Aortic distensibility (D) was computed as a downstream application of the segmentation results from the ascending (AAo) and descending (DAo) aorta across the cardiac cycle [49]. Specifically, aortic distensibility is calculated using the area changes in the aorta over the cardiac cycle, based on the segmented cross-sectional areas. The formula used for calculation is as follows:

(5) $$D=\frac{A_{max}-A_{min}}{A_{min}\times CPP}\times 10^{3},$$

where $A_{max}$ and $A_{min}$ are the maximum and minimum aortic areas, and *CPP* (central pulse pressure) is expressed in mmHg. Subjects with *CPP* < 10 mmHg or failed quality checks were excluded from analysis.

Aortic distensibility is calculated as a downstream validation task for the segmentation performance of the cardiac structures, particularly the aortic regions. By leveraging the accurate segmentation of the aorta, we were able to compute aortic distensibility, which demonstrates the clinical relevance of the segmentation model. This task is not intended as a standalone clinical application, but as a validation of the segmentation framework, showing that reliable and precise segmentation of the cardiac structures enables the computation of clinically valuable parameters, such as aortic distensibility.

In other words, the primary focus of the analysis is not to estimate distensibility in isolation but to validate the segmentation process by showing that accurate segmentations of the aortic cross-sections can provide clinically useful measurements. This also supports the utility of the proposed segmentation framework for real-world clinical applications, emphasizing its potential in downstream tasks.

### 2.8. Loss Function and Optimization for MI Segmentation

To address the pronounced class imbalance inherent in MI segmentation, where infarct regions occupy only a small fraction of the image area, a composite loss function combining Dice loss and Focal loss was employed. This combination balances spatial overlap accuracy with robust learning from hard-to-classify pixels.

The Dice loss directly optimizes spatial overlap between the predicted segmentation and the ground truth and is defined as:

(6) $$DiceLoss=1-\frac{2\left|y_{true}\cap y_{pred}\right|+\epsilon }{\left|y_{true}\right|+\left|y_{pred}\right|+\epsilon }$$

where $y_{\mathrm{true}}$ denotes the ground truth binary mask, $y_{\mathrm{pred}}$ represents the predicted probability map output by the network, ∣⋅∣ indicates the sum over all pixels, and ϵ is a small constant added for numerical stability.

To further mitigate the impact of severe foreground–background imbalance and emphasize difficult pixels, Focal loss was incorporated. It is defined as:

(7) $$\begin{array}{c} FocalLoss=-\alpha {\left(1-y_{pred}\right)}^{\gamma }{\times y}_{true}\;\times log(y_{pred})\\ \;-\left(1{-y}_{true}\right){\;\times \left(1-y_{pred}\right)}^{\gamma }\times \;log\left(1-y_{pred}\right) \end{array}$$

where α is a weighting factor that balances the contribution of infarct and background classes, γ is the focusing parameter that reduces the relative loss contribution from easy examples, and $y_{\mathrm{true}}\in (0,1)$ and $y_{\mathrm{pred}}\in (0,1)$.

The final loss function is defined as a weighted combination of Dice and Focal losses:

(8) $$TotalLoss=\left(0.5\times DiceLoss\right)+\left(0.5\times FocalLoss\right)$$

The Attention U-Net model for MI segmentation was optimized using the Adam optimizer with a learning rate of $10^{-4}$. Early stopping was applied with a patience of 5 epochs based on validation loss, and adaptive learning rate reduction was employed with a reduction factor of 0.5 and a patience of 3 epochs to improve convergence stability and prevent overfitting.

### 2.9. Evaluation Metrics

Model performance was evaluated using complementary segmentation and pixel-level classification metrics. For anatomical structure segmentation, spatial overlap was assessed using the Dice Similarity Coefficient (DSC), and boundary accuracy was quantified using the Mean Contour Distance (MCD) and the Hausdorff Distance (HD) [50].

Together, these metrics characterize both regional agreement with expert annotations and boundary delineation quality.

For myocardial infarction (MI) segmentation, where infarct pixels are sparse and class imbalance is severe, evaluation emphasized Dice, precision, recall, and F1-score, which are more informative than overall pixel accuracy in this setting. A pixel-level confusion matrix was also computed to summarize true positives (TP), false positives (FP), true negatives (TN), and false negatives (FN).

To ensure transparent and reproducible evaluation, the Attention U-Net produces a continuous probability map p(x)∈[0,1]. For segmentation metrics (Dice, precision, recall, and F1), this probability map was converted to a binary infarct mask using a fixed threshold of 0.5 (i.e., pixels with p(x)≥0.5 were labeled as infarct). In contrast, ROC–AUC was computed directly from the unthresholded probability outputs by sweeping the decision threshold over [0, 1], and therefore does not depend on a single operating point.

The DSC is defined as:

(9) $$DSC(P,\;G)\;=\;\frac{2\left|P\cap G\right|}{\left|P\right|+\left|G\right|}$$

where P denotes the predicted foreground set (segmented region), and G represents the ground truth foreground set.

Pixel-wise classification metrics were computed from *TP*, *FP*, *TN*, and *FN*, as follows:

(10) $$\begin{array}{c} Precision\;=\;\frac{TP}{TP\;+\;FP},\;\;\;\;\;\;\;\;\;\;\;Recall\;=\;\frac{TP}{TP\;+\;FN}\;\;\;\;\;\;\;\;\;\\ F1\;=\;\frac{2(Precision\;\times \;Recall)}{Precision\;+\;Recall}\;,\;\;\;\;\;\;\;\;Accuracy\;=\;\frac{TP+TN}{TP\;+\;TN\;+\;FP+FN} \end{array}$$

To quantify boundary accuracy, let ∂P and ∂G denote the predicted and ground truth contours, respectively. The point-to-set distance from a contour point x to ∂G is defined as $d(x,\partial G)={min}_{y\in \partial G}∥x-y∥$. The *MCD* was computed as a symmetric average surface distance:

(11) $$MCD(\partial P,\;\partial G)\;=\frac{1}{2}\;\left(\frac{1}{\left|\partial P\right|}\sum_{x\in \partial P}d(x,\;\partial G)+\frac{1}{\left|\partial G\right|}\sum_{y\in \partial G}d(y,\;\partial P)\right)$$

*HD* measures the maximum boundary discrepancy and is defined as:

(12) $$HD(\partial P,\;\partial G)\;=max\left\{\underset{x\in \partial P}{\max}d(x,\;\partial G),\;\underset{y\in \partial G}{\max}d(y,\;\partial P)\right\}\;$$

For MI detection, the ROC curve was generated by varying the decision threshold, and the Area Under the ROC Curve (AUC) was computed as the integral of the ercall versus the False Positive Rate (*FPR*):

(13) $$Recall\;=\;\frac{TP}{TP\;+\;FN},\;\;\;\;\;\;\;\;\;\;\;FPR\;=\;\frac{FP}{FP\;+\;TN}$$

## 3. Results

### 3.1. Overview of Research Objectives

This study investigates deep learning approaches for two primary tasks in cardiac MRI analysis. The first objective was to automate the segmentation of key anatomical structures, including the LV cavity, LV myocardium, and RV cavity, through the use of multiple CNN architectures. The second objective focused on the localization of MI using an attention-augmented U-Net, enhanced with SE blocks. The results are presented according to these two objectives and are evaluated through visual comparisons, quantitative metrics, and classification accuracy.

### 3.2. Cardiac Structure Segmentation Performance

#### 3.2.1. Visual Segmentation Outputs and Structural Validation

All proposed CNN variants successfully delineated cardiac structures across temporal frames of the cine MRI sequence. Visual inspection confirms consistent spatial alignment between the predicted contours and anatomical boundaries across different cardiac phases and patients. To ensure transparent qualitative validation, the network-generated segmentations are presented alongside expert manual (ground truth) annotations, with all visual comparisons performed on the same cine MRI slices. These qualitative results are intended to complement, rather than replace, the quantitative segmentation scores provided later in Section 3.2.6.

**Figure 2:**
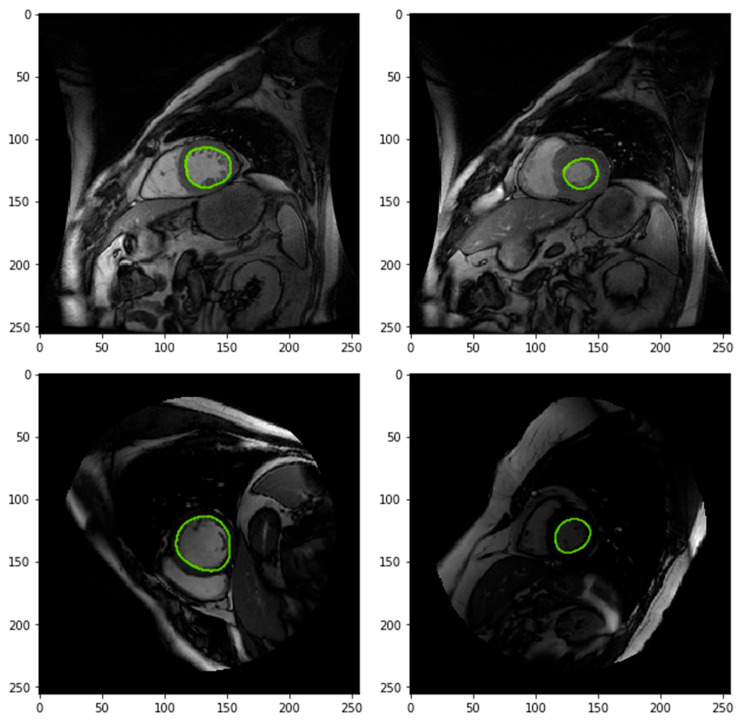
CNN-predicted cardiac structure segmentation across the cardiac cycle. Representative cine CMR slices at different cardiac phases with CNN-predicted contours overlaid in green. The contours demonstrate consistent tracking of ventricular shape and boundary deformation across time. High-contrast visualization and improved resolution are used to ensure clear visibility of the segmented regions, including challenging apical slices.

Figure 2 illustrates representative cine CMR slices at different cardiac phases, with CNN-predicted contours overlaid using high-contrast coloring. Enhanced visualization quality allows for clear identification of the segmented regions and demonstrates the ability of the proposed models to track cardiac motion and structural deformation throughout the cardiac cycle. In most cases, the predicted contours closely follow the anatomical boundaries of the ventricular structures. Challenging examples, including difficult apical slices and cases of partial failure, are intentionally included to highlight scenarios where boundary localization remains more ambiguous.

**Figure 3:**
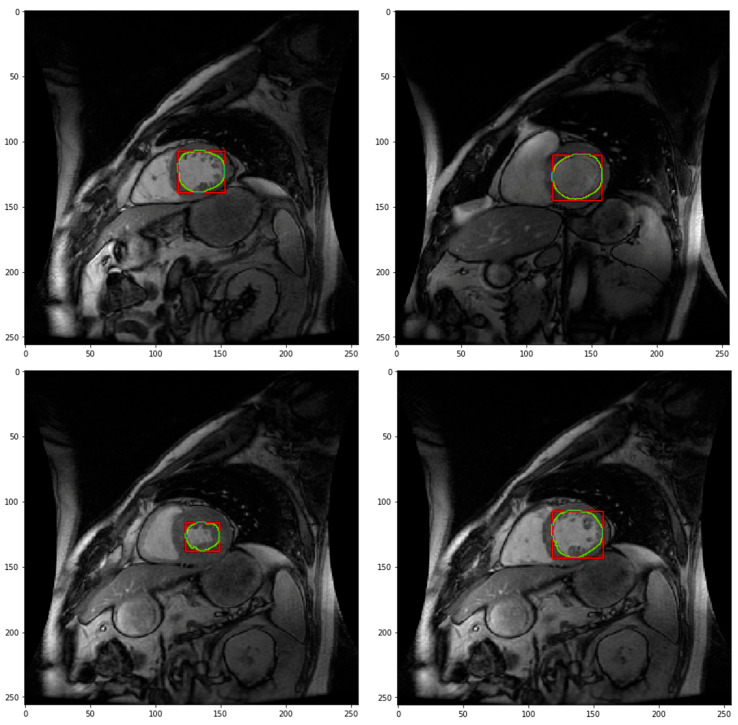
Localization and ROI validation for cardiac structure segmentation. Predicted segmentation contours (green) shown within automatically extracted region-of-interest (ROI) bounding boxes (red) for representative slices. The consistent centering of contours within the ROIs confirms reliable localization and spatial consistency of the CNN-based segmentation framework.

Figure 3 presents predicted segmentation contours overlaid within automatically extracted region-of-interest (ROI) bounding boxes. The predicted contours (green) are consistently centered within the ROIs (red), confirming reliable localization and spatial consistency of the CNN-based segmentation across slices and subjects. This visualization further validates the robustness of the ROI extraction and localization stage, which precedes fine-grained segmentation.

**Figure 4:**
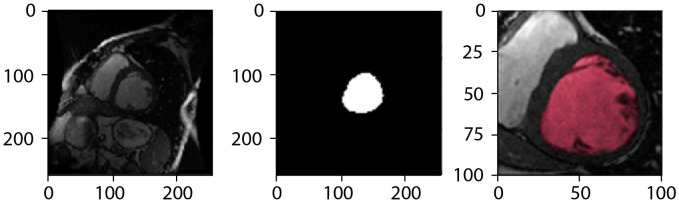

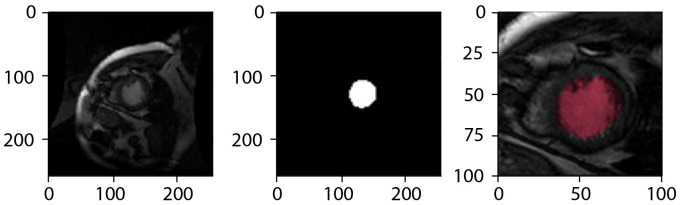

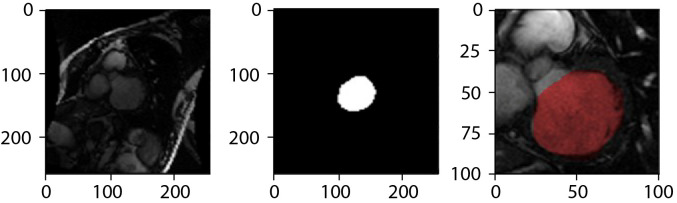
Qualitative comparison between network-generated and manual segmentations. Representative examples showing, for each row, the same cine MRI slice presented as (**left**) the original input image, (**middle**) the binary segmentation mask generated by the CNN-based cardiac structure segmentation model, and (**right**) the corresponding expert-annotated ground truth segmentation. In the middle column, the mask has been overlaid on the original image to enhance visibility and demonstrate correspondence. The right column shows the zoomed-in ground truth segmentation with the infarcted region highlighted in red to improve contrast. The side-by-side layout enables direct visual comparison of boundary agreement, shape fidelity, and anatomical consistency between predicted and manual segmentations.

Figure 4 provides explicit qualitative validation by presenting, for each row, the same cine MRI slice in three corresponding representations: (left) the original input image, (middle) the binary segmentation mask generated by the CNN-based cardiac structure segmentation model, and (right) the expert-annotated ground truth segmentation for that slice. The middle-column masks correspond to the direct outputs of the best-performing CNN variant identified in the architectural comparison experiments, without additional post-processing beyond the standard pipeline described in Section 2.

To enhance clarity, we have overlaid the binary segmentation mask directly onto the original MRI images (left) and the zoomed-in images (right), where the infarcted region is highlighted in red. This side-by-side presentation enables direct visual comparison between network-generated and manual segmentations, allowing for assessment of boundary agreement, shape fidelity, and anatomical plausibility. Across diverse subjects and cardiac phases, the predicted segmentations show strong visual correspondence with the ground truth, with residual discrepancies primarily observed in anatomically challenging regions such as the basal and apical slices. Quantitative segmentation accuracy for the LV cavity, LV myocardium, and RV cavity—aggregated across the full test cohort and all evaluated architectures—is reported in Section 3.2.6. By explicitly pairing predicted and manual masks for the same slices, this section clarifies the relationship between the columns in Figure 4 and provides transparent visual validation of the proposed framework.

#### 3.2.2. Architectural Variants and Ground Truth Comparison

Segmentation performance was compared across four distinct CNN architectures—shallow, deeper, larger, and max-pooling—to systematically evaluate the impact of architectural design choices on cardiac structure segmentation. In addition, a ReLU-activated configuration of the shallow CNN was examined to isolate the effect of activation function choice under otherwise identical architectural and training conditions. This ReLU configuration does not constitute a separate network architecture; rather, it represents a parameter-level variant introduced solely to assess optimization and representation behavior. Quantitative segmentation performance for all anatomical structures and evaluated architectures is summarized in Section 3.2.6.

The qualitative differences correspond to visible variations in boundary smoothness, regional continuity, and spatial alignment relative to the ground truth. The deeper and ReLU-activated variants exhibit fragmented or partially missing masks in this slice, whereas the larger and max-pooling architectures retain more coherent region structure and improved boundary adherence. These trends are consistent with the superior average performance of the latter architectures reported quantitatively.

To formally evaluate architectural differences, statistical testing of per-case Dice scores across the full dataset was performed using paired t-tests. Because seven pairwise comparisons were conducted, the Holm–Bonferroni correction was applied to control the family-wise error rate (FWER) at α = 0.05. This sequential adjustment procedure ranks *p*-values from smallest to largest and applies progressively stricter significance thresholds to mitigate inflation of Type I error due to multiple testing.

Table 4 presents the nominal *p*-values for each comparison together with their significance status after Holm–Bonferroni correction.

The results show that the comparisons between Shallow vs. Larger, Shallow vs. Deeper, and Larger vs. ReLU remain statistically significant after Holm–Bonferroni correction, with nominal *p*-values below their respective adjusted thresholds. In contrast, comparisons such as Shallow vs. Max-pooling, Larger vs. Max-pooling, and Larger vs. Deeper did not retain statistical significance once multiple testing was accounted for, indicating that these architectural differences do not yield robust performance improvements under family-wise error control.

This corrected statistical analysis ensures that reported architectural differences are not driven by chance due to repeated testing. When considered together with the region-wise Dice, MCD, and HD values reported in Section 3.2.6, the results provide a rigorous and transparent assessment of segmentation performance across the left ventricle cavity, left ventricular myocardium, and right ventricle cavity.

#### 3.2.3. Optimization Dynamics and Loss Sensitivity

**Figure 5:**
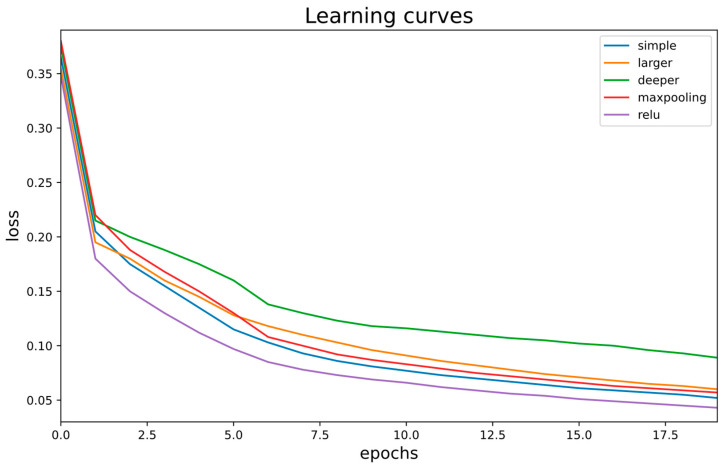
Training convergence behavior across CNN architectures. Loss convergence curves (mean squared error) over 20 training epochs for five CNN configurations: shallow (simple), larger, deeper, max-pooling, and a ReLU-activated shallow variant. All models exhibit stable, monotonic loss reduction, indicating consistent optimization under identical training conditions. Differences in convergence rate and final loss highlight the impact of architectural design choices on optimization efficiency, with shallower and ReLU-activated configurations converging more rapidly than deeper variants.

To assess training stability and convergence behavior, we monitored the mean squared error (MSE) loss over 20 epochs for five CNN variants: shallow (simple), larger, deeper, max-pooling, and a ReLU-activated shallow configuration. As shown in Figure 5, all architectures exhibit a monotonic decrease in loss, confirming stable optimization under identical training conditions. However, clear differences are observed in both convergence rate and final loss values.

The ReLU-activated and shallow (simple) architectures converge more rapidly and reach lower final loss values, indicating more efficient optimization dynamics [51]. In contrast, the deeper network converges more slowly and plateaus at a higher loss level, suggesting increased sensitivity to optimization dynamics and a higher likelihood of suboptimal convergence as network depth increases. The larger and max-pooling variants demonstrate intermediate behavior, reinforcing that increased architectural complexity does not necessarily translate into improved convergence characteristics for cine CMR segmentation.

Although the loss curves suggest that additional training epochs could yield further marginal reductions in loss, preliminary observations showed that the rate of improvement beyond 20 epochs becomes progressively smaller. Given the limited size of the training dataset and the ROI-based regression formulation adopted in this study, further extension of training increased the risk of overfitting without providing significant gains in generalization. For this reason, a fixed training duration of 20 epochs was selected to balance convergence, robustness, and fair comparison across architectures. Importantly, all CNN variants were trained for the same number of epochs under identical optimization settings, ensuring that observed performance differences are attributable to architectural design rather than training duration.

**Figure 6:**
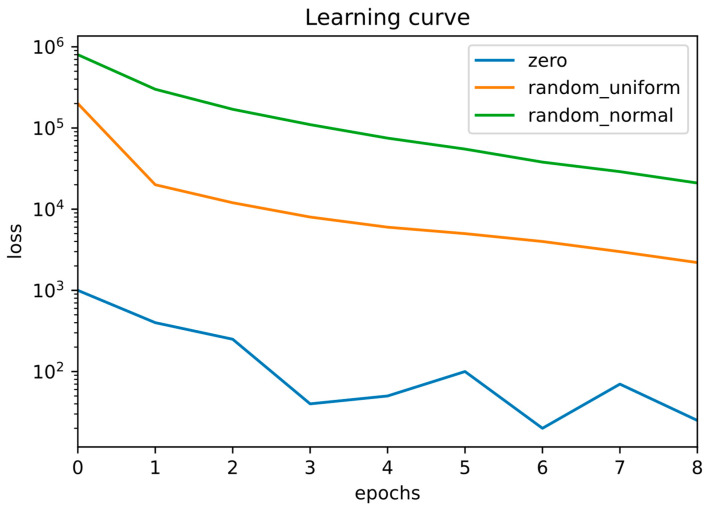
Effect of weight initialization on training loss dynamics. Training loss (mean squared error) trajectories over 20 epochs for three weight initialization strategies—zero, random uniform, and random normal—under identical network architecture, optimization, and training conditions. All curves exhibit stable loss decay, while zero initialization shows faster early-stage convergence compared to random initialization schemes. The figure illustrates optimization dynamics only and is not intended to represent segmentation accuracy.

We further examined the effect of weight initialization strategies by comparing zero, random_uniform, and random_normal initialization under otherwise identical training conditions. As illustrated in Figure 6, zero initialization resulted in faster loss reduction and lower loss values during early training compared to both random initialization schemes for the CNN architectures evaluated in this study.

Importantly, Figure 6 reports training loss (MSE). Dice metrics were evaluated after training convergence and are reported separately in Section 3.2.6, consistent with standard practice in segmentation studies, where overlap metrics are assessed on fully trained models rather than during optimization.

While classical deep learning theory discourages zero initialization due to symmetry-breaking concerns, it is crucial to emphasize that the architectures used here are shallow, strongly regularized, and trained on small, localized regions of interest (ROIs) rather than full-resolution images. In this specific setting, zero initialization provides a stable and uniform starting point that does not hinder optimization. This is likely because symmetry is rapidly broken by nonlinear activations, bias terms, and data-driven gradient updates. Similar behavior has been reported in compact or regression-based segmentation networks operating on constrained input domains.

We note that the initialization comparison presented in Figure 6 represents an exploratory analysis conducted using a single training run per initialization scheme under a fixed random seed. While multiple independent runs would be required to draw statistically robust conclusions regarding initialization sensitivity, the purpose of this analysis is to illustrate qualitative differences in early optimization behavior under identical conditions, rather than to establish definitive performance rankings.

Accordingly, these observations should be interpreted within the specific architectural depth, loss formulation, and ROI-based regression setting adopted in this study. Under this configuration, zero initialization exhibited more stable early convergence than the evaluated random initialization schemes, highlighting that initialization behavior depends on architectural design and data regime, particularly in shallow segmentation networks trained on limited datasets.

To further assess the influence of the optimization objective, we performed a controlled loss-function sensitivity analysis using the same network architecture, data split, and training protocol. The model was trained separately with four loss formulations: mean squared error (MSE), binary cross-entropy (BCE), Dice loss, and a combined BCE+Dice loss, while keeping all other hyperparameters fixed to ensure a fair comparison.

The results, summarized in Table 5 show that Dice-based formulations achieved higher overlap metrics, with the BCE+Dice configuration yielding the highest Dice score (0.8184) and IoU (0.7104). The MSE formulation produced slightly lower overlap performance (Dice = 0.7740, IoU = 0.6508), while remaining comparable to BCE.

Although Dice-based losses maximize overlap metrics, MSE was retained for the architectural benchmarking experiments for methodological consistency. The mask-level regression formulation benefits from continuous-valued prediction maps, which encourage smoother contour representations under weak cine contrast conditions. In addition, the stacked autoencoder refinement stage operates within a reconstruction framework that is naturally aligned with regression-based objectives. Maintaining a consistent loss formulation across CNN variants therefore ensured controlled architectural comparison without introducing additional optimization variables.

Overall, the loss comparison indicates that while Dice-based objectives improve overlap metrics, the performance differences are moderate, and the regression-based formulation remains stable and compatible with the integrated segmentation and refinement pipeline.

#### 3.2.4. SAE Post-Processing, Ablation, and Loss Comparison

In this study, a three-stage stacked autoencoder (SAE) was employed as a post-processing module to enhance the anatomical accuracy and spatial coherence of the initial CNN-generated segmentation masks. This refinement is particularly beneficial in cine CMR, where myocardial boundaries are often weakly defined and segmentation errors tend to concentrate in anatomically variable basal and apical slices. The SAE stage was designed to suppress fragmentation, correct topological inconsistencies, and improve global mask plausibility prior to final boundary regularization.

Although the SAE module is well described, it is important to isolate its contribution relative to the subsequent active contour stage. To clarify the individual effects of each refinement component, we conducted an ablation analysis comparing three configurations:

- CNN-only predictions;
- CNN followed by SAE refinement;
- CNN followed by SAE refinement and active contour regularization.

The quantitative results are summarized in Table 6.

The SAE refinement stage yields the largest improvement in regional overlap, increasing Dice from 0.66 to 0.76, and substantially reducing boundary deviation (HD: 80 mm to 44 mm). This indicates that SAE primarily enhances global mask coherence and reduces structural fragmentation.

The addition of active contour regularization produces a smaller but consistent Dice improvement (0.76 to 0.79), while markedly decreasing the Hausdorff Distance (44 mm to 26 mm). This behavior is consistent with the role of active contour models as boundary refinement mechanisms that suppress local contour outliers and improve worst-case boundary alignment.

Together, these results demonstrate that SAE and active contour provide complementary benefits: SAE strengthens anatomical plausibility at the structural level, while active contour further refines boundary precision.

To evaluate the effect of different optimization strategies within the SAE framework, three SAE loss configurations were examined:

- Customized loss combined with MSE;
- Symmetric MSE applied to both encoder and decoder stages;
- KL divergence combined with MSE.

**Figure 7:**
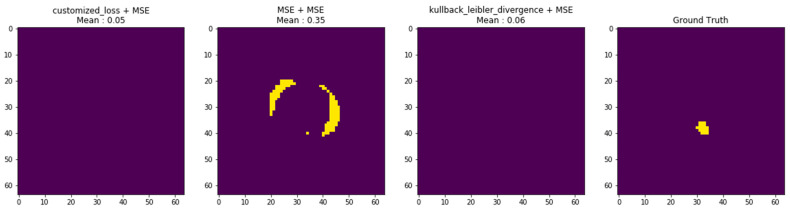
Visual comparison of SAE-refined segmentations using different loss configurations. The left-to-right panels show (1) Customized loss + MSE, (2) MSE + MSE, (3) KL divergence + MSE, and (4) Ground truth mask. The MSE + MSE configuration yielded the most accurate and complete segmentation results, as evidenced by improved boundary coherence and reduced fragmentation.

Figure 7 presents a qualitative comparison of the refined segmentation outputs under these loss formulations.

Before SAE refinement, the CNN-only segmentation exhibited limited structural coherence (Dice = 0.046; HD = 90.8 mm) in this challenging example, reflecting the difficulty of segmenting weak-contrast myocardial regions in cine CMR. After SAE refinement using the symmetric MSE formulation, Dice improved to 0.345 and HD decreased to 33.7 mm, indicating substantial recovery of spatial consistency and contour continuity.

Although the refined Dice value does not represent final pipeline performance, the observed improvements (ΔDice and ΔHD) confirm that SAE effectively corrects fragmentation and enforces anatomically plausible mask topology prior to contour regularization.

The quantitative comparison across SAE loss settings is summarized in Table 7. The symmetric MSE configuration achieved the most stable and anatomically coherent refinement, suggesting that regression-consistent objectives promote effective contour encoding and decoding within the SAE reconstruction framework.

The Correlation Coefficient (CC) reported in Table 7 was specifically designed to evaluate boundary alignment between predicted and reference contours. Unlike classical Pearson correlation (−1 to 1), which measures intensity relationships, the CC used here quantifies geometric similarity in contour shape and spatial positioning. Higher positive values indicate stronger boundary agreement, while negative values may occur in cases of pronounced contour misalignment or topological inconsistencies (e.g., partial inversion or displacement). In this context, CC serves as an indicator of structural consistency rather than numerical instability.

#### 3.2.5. Final Contour Recovery

A post-processing pipeline incorporating SAEs and active contour modeling was employed to enhance the anatomical precision of CNN-predicted segmentations. This pipeline refines the initial binary masks by learning a regularized shape representation through the SAE and subsequently enforces boundary smoothness using deformable contour evolution, addressing irregularities and spurious predictions commonly observed in raw CNN outputs.

Active contour modeling, also referred to as deformable models or “snakes,” is applied as a deterministic, energy-minimization step that evolves an initial contour toward anatomically meaningful boundaries. In this work, the initial contour is derived from the SAE-refined segmentation mask and iteratively updated by minimizing an energy functional composed of internal and external terms. The internal energy enforces contour smoothness and continuity, while the external energy attracts the contour toward image-derived features, such as intensity gradients at tissue boundaries. This formulation allows the contour to adapt to local image structure while preserving global shape regularity.

Importantly, the active contour stage does not perform learning or classification. Instead, it operates as a geometry-based refinement mechanism that corrects minor boundary inaccuracies, fills small gaps, and suppresses jagged edges introduced by pixel-wise segmentation. This is particularly beneficial in cine CMR images, where myocardial borders can be weakly defined and affected by motion, noise, or partial-volume effects.

**Figure 8:**
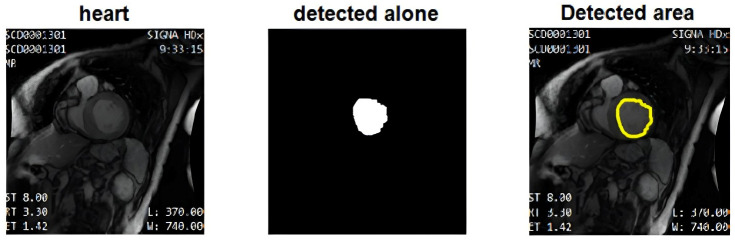
Stepwise refinement. From left to right: (1) Original MRI slice, (2) CNN-derived binary mask of the segmented region, (3) Final refined contour (yellow) overlaid on the original image after post-processing. This figure demonstrates the improvement in boundary smoothness and anatomical coherence achieved through SAE-based refinement and active contour modeling.

Figure 8 illustrates the contour refinement process, from the initial raw segmentation to the final output. The first panel shows the original MRI slice, the second panel shows the CNN-derived binary mask, and the third panel overlays the final contour obtained after SAE-based regularization and active contour evolution (yellow) on the original image.

**Figure 9:**
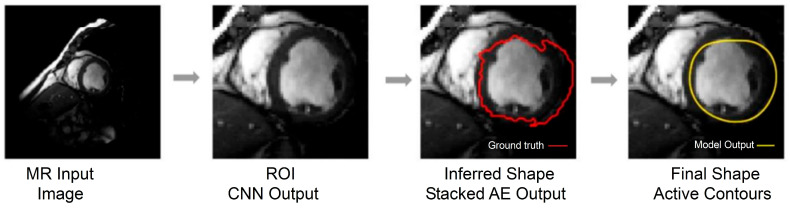
Full segmentation pipeline. Overlay of ground truth annotations (red) alongside snake post-processing results (yellow) to verify that the contour movement aligns with the ground truth. From left to right: (1) Original MRI input, (2) CNN-predicted region of interest (ROI), (3) SAE-based inferred shape representation (red contour, ground truth annotation), and (4) Final active contour output (yellow contour, model output). This figure demonstrates the end-to-end segmentation workflow, highlighting the role of SAE and active contour modeling in producing smooth and anatomically plausible final contours.

Figure 9 provides a visual overview of the complete segmentation pipeline. From left to right, it shows the original MRI input, the CNN-predicted region of interest (ROI), the SAE-refined shape representation (red contour, ground truth), and the final active contour output (yellow contour, model output). The ROI shown in Figure 9 is generated automatically from the CNN prediction by computing the bounding box of the foreground region in the CNN-derived mask, applying a fixed padding margin, and cropping the corresponding image region. This ROI extraction is fully prediction-driven and does not use ground truth information at inference time. The cropped ROI is subsequently used as input for SAE-based shape refinement and active contour optimization. The red contour represents the ground truth annotation, and the yellow contour represents the model output (active contours) after post-processing.

This sequence demonstrates how prediction-driven ROI localization, followed by SAE-based shape regularization and active contour evolution, produces smooth and anatomically plausible final segmentations. The close alignment between the refined contours and expert annotations highlights the effectiveness of combining learning-based segmentation with classical deformable modeling to improve boundary accuracy under weak-contrast and complex anatomical conditions.

#### 3.2.6. Segmentation Accuracy Metrics

Table 8 presents the segmentation performance of the final output, achieved after convolutional CNN prediction followed by SAE refinement and active contour post-processing. Performance is evaluated using complementary overlap- and boundary-based metrics, including the DSC and HD. These metrics offer a comprehensive assessment of both regional agreement and boundary accuracy.

High Dice scores across all anatomical regions indicate strong volumetric agreement between the final predicted segmentations and expert annotations. However, boundary-based evaluations reveal comparatively higher HD values for the RV cavity. This finding is anatomically expected, as the RV exhibits a thin-walled, crescent-shaped geometry with significant inter-slice variability and less well-defined boundaries in cine cardiovascular CMR, particularly in the basal and apical slices. Since HD measures the maximum boundary deviation, localized mismatches along the RV free wall or apex can lead to higher HD values, despite maintaining high global overlap. Therefore, the combination of high Dice scores and elevated HD reflects localized boundary sensitivity rather than systematic segmentation failure.

**Table 8:** Segmentation performance of the final post-processed model per anatomical region.

Region	Group	Dice (DSC)	HD (mm)
LV cavity	Full Test	0.93 ± 0.05	3.28 ± 1.02
LV myocardium	Full Test	0.89 ± 0.04	4.05 ± 1.45
RV cavity	Full Test	0.91 ± 0.06	7.40 ± 2.75
LV cavity	CVD Cases	0.95 ± 0.04	3.75 ± 1.22
LV myocardium	CVD Cases	0.88 ± 0.05	4.42 ± 1.28
RV cavity	CVD Cases	0.89 ± 0.04	8.32 ± 3.04

To evaluate the robustness of the framework across patient populations, results are presented separately for the full test cohort and the cardiovascular disease (CVD) subgroup. Comparable performance trends across both groups demonstrate the framework’s stability under varying anatomical and pathological conditions.

In addition to region-level segmentation accuracy, pixel-level classification performance was assessed for the final post-processed segmentation output. Specifically, recall was computed on a pixel-wise basis, treating the segmentation task as a binary foreground-background classification problem across all voxels from 805 test volumes. The reported overall recall of 0.959 quantifies the sensitivity of the final segmentation pipeline in correctly identifying foreground (anatomical structure) pixels across the entire test set, rather than focusing on a specific anatomical region or subgroup.

**Figure 10:**
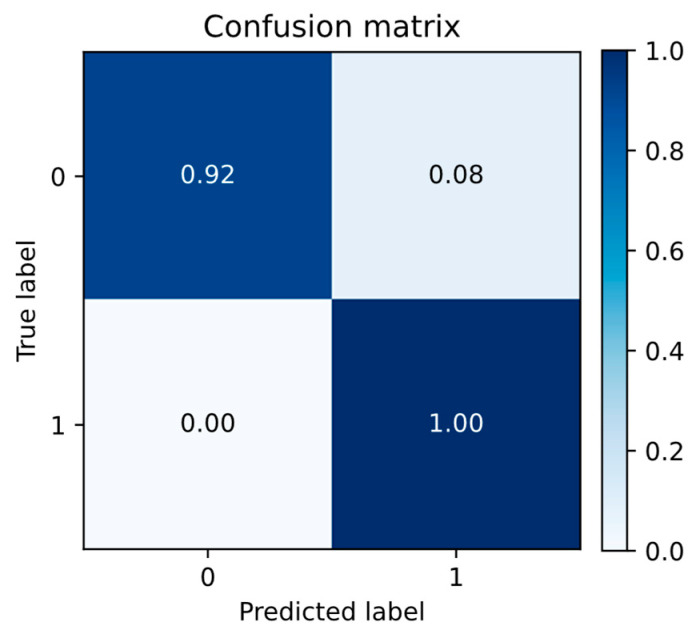
Pixel-level confusion matrix for the final segmentation output. Normalized confusion matrix computed over all pixels from 805 test volumes, treating segmentation as a binary foreground–background classification task. The resulting recall of 0.959 reflects the sensitivity of the final segmentation pipeline to correctly identify anatomical structure pixels across the full test set.

The corresponding normalized confusion matrix shown in Figure 10 summarizes the pixel-level evaluation, confirming high sensitivity for foreground detection and high specificity for background classification. Together with the region-wise metrics, these results demonstrate that the proposed framework achieves both accurate anatomical delineation and reliable pixel-level detection.

### 3.3. MI Segmentation Performance

#### 3.3.1. Visual Results and Heatmap Inference

MI segmentation was evaluated on a test set comprising 75 infarct-labeled cardiac MRI slices.

**Figure 11:**
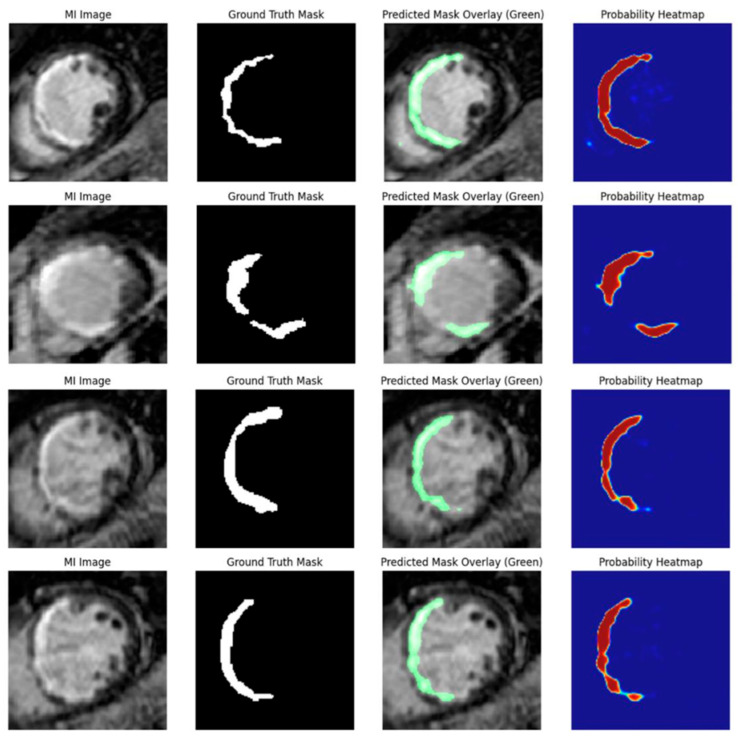
Contrast-free myocardial infarction segmentation from cine CMR. Representative examples showing, from left to right, the input cine MRI slice, the expert-annotated ground-truth infarction mask, the predicted infarct segmentation overlaid on the input image (green), and the corresponding pixel-wise SoftMax probability heatmap. Warmer colors in the heatmap indicate higher model confidence for infarct tissue, illustrating accurate spatial localization and boundary delineation without contrast enhancement.

All MI detection and segmentation results are reported at the slice level, with each cine MRI frame treated as an independent sample. Representative outputs from the proposed Attention U-Net model enhanced with squeeze-and-excitation (SE) blocks are shown in Figure 11. From left to right, the figure presents the original MI image, the corresponding expert-annotated ground truth mask, the predicted segmentation overlaid on the input image (green), and the pixel-wise SoftMax probability heatmap.

The predicted infarct regions show strong spatial correspondence with the ground truth across diverse infarct morphologies and anatomical locations. The probability heatmaps visualize model confidence at the pixel level, with red and orange indicating high infarct likelihood and blue representing background or low-confidence regions. Together, these visualizations demonstrate the model’s ability to localize and delineate myocardial infarction with high anatomical fidelity using contrast-free cine MRI.

#### 3.3.2. Classification Metrics and ROC Analysis

To further quantify myocardial infarction (MI) segmentation performance, overlap-based and threshold-independent metrics were emphasized, given the pronounced foreground–background imbalance inherent in pixel-wise infarct detection. In this setting, overall pixel accuracy may be artificially inflated by the predominance of background pixels and is therefore not considered a primary indicator of segmentation quality.

Using a fixed probability threshold of 0.5 to derive binary masks from the predicted probability maps (as described in Section 2.9), the proposed Attention U-Net achieved a Dice score of 0.77 and a recall of 0.80 on the test set comprising 75 infarct-labeled cine CMR slices. While this recall indicates that the majority of infarct pixels were correctly identified, approximately 20% were missed. In clinical contexts, such false negatives may lead to underestimation of infarct extent, particularly in small, diffuse, or subendocardial lesions.

To better characterize robustness under the limited sample size (*n* = 75), variability was estimated using bootstrap resampling (1000 iterations). The resulting 95% confidence intervals are summarized in Table 9. The Dice score demonstrated moderate variability (95% CI: 0.69–0.84), consistent with the intrinsic difficulty of contrast-free infarct delineation. Recall and precision exhibited comparable stability ranges, supporting the reliability of the reported performance estimates.

The observed variability partly reflects the inherent challenges of infarct delineation in non-contrast cine CMR. Unlike late gadolinium enhancement imaging, cine MRI does not provide direct scar-specific contrast; infarcted regions often manifest as subtle motion abnormalities or intensity variations, making precise pixel-level segmentation intrinsically challenging.

**Figure 12:**
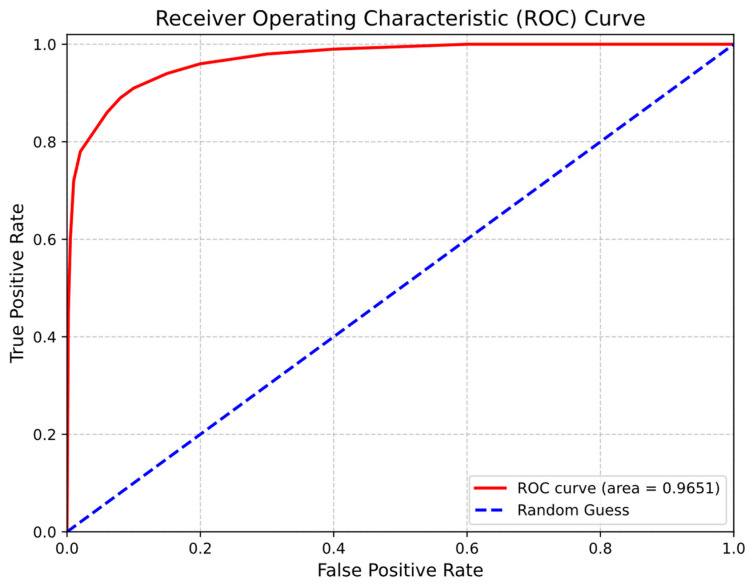
Receiver Operating Characteristic (ROC) curve for myocardial infarction classification on 75 infarct-labeled slices. The model achieved an area under the curve (AUC) of 0.9651, demonstrating strong discriminative ability between infarcted and non-infarcted tissue.

Figure 12 presents the Receiver Operating Characteristic (ROC) curve, illustrating the trade-off between recall and false positive rate across varying decision thresholds. The model achieved an area under the curve (AUC) of 0.9651, indicating strong discriminative capability between infarcted and non-infarcted myocardial tissue. Because AUC is computed from the continuous probability outputs without fixing a threshold, it provides a threshold-independent assessment of ranking performance. The ROC curve remains well above the random baseline across thresholds, confirming robust discriminative behavior.

The operating threshold used to report Dice and recall was selected to balance sensitivity and specificity rather than to maximize sensitivity alone. Depending on the intended clinical application, alternative thresholds favoring higher sensitivity may be adopted.

Overall, these results demonstrate that the proposed model achieves strong overlap-based performance and robust discriminative capability for MI detection in contrast-free cine CMR, while the reported confidence intervals provide a transparent assessment of robustness under limited-data conditions.

## 4. Discussion

This study presents a unified deep learning framework for automatic segmentation of cardiac structures and myocardial infarction (MI) using contrast-free cine CMR. While prior work has demonstrated the effectiveness of deep learning for cardiac image segmentation and infarct analysis, several challenges remain unresolved, including limited generalizability across heterogeneous datasets, reliance on contrast-enhanced imaging, and reduced boundary accuracy in anatomically variable regions. The proposed framework addresses these challenges by integrating controlled CNN architectural benchmarking, attention-based MI segmentation, and stacked autoencoder-based contour refinement within a single, cohesive pipeline. This work emphasizes robustness in contrast-free settings, with a focus on demonstrating feasibility under realistic data constraints rather than replacing contrast-enhanced protocols.

Across the four CNN architectures evaluated, the framework achieved robust cardiac structure segmentation, with Dice similarity coefficients of 0.93 ± 0.05 for the LV cavity, 0.89 ± 0.04 for the LV myocardium, and 0.91 ± 0.06 for the RV cavity. These results are comparable to, and in some cases slightly exceed, previously reported benchmarks such as those by Bai et al. [10] and Ammar et al. [52], despite the absence of pre-cropped regions of interest or task-specific preprocessing. This demonstrates the scalability and resilience of the proposed approach across architectural variations and imaging conditions.

A distinctive design choice in this work is the use of a dense output layer for segmentation, framing the task as mask-level regression rather than fully convolutional pixel-wise prediction. Although less conventional, this formulation enables explicit encoding of global anatomical shape while preserving local spatial detail, which is particularly important in cine CMR where myocardial boundaries are often weakly defined [53,54]. In this setting, CNN architecture primarily determines the quality of the initial localization, while subsequent post-processing stages refine boundary geometry without altering learned image features.

For myocardial infarction localization, the Attention U-Net augmented with squeeze-and-excitation (SE) blocks achieved a pixel-level accuracy of 0.98 ± 0.01 and a recall of 0.80 ± 0.02 using non-contrast cine CMR. Given the limited number of infarct-positive slices (75), these results are intended to demonstrate feasibility under real-world data constraints rather than claim definitive clinical performance.

Recent work on contrast-free infarct analysis has largely focused on cine-to-LGE or cine-to-enhancement generative approaches, including virtual native enhancement (VNE), cine-generated enhancement (CGE), and diffusion-based synthesis frameworks [31,32,33,34]. These methods aim to reconstruct contrast-like images from cine or multi-sequence inputs, enabling visualization and quantification of scar tissue without gadolinium administration. While promising, these methods primarily focus on image synthesis and enhancement quality, rather than direct optimization of segmentation robustness and anatomical boundary fidelity. In contrast, this framework performs infarct localization directly on native cine CMR, evaluating how architectural design choices and post-processing strategies influence segmentation accuracy, particularly in anatomically challenging regions.

The studies summarized in Table 10 were conducted using different datasets, imaging protocols, annotation standards, and evaluation strategies. Accordingly, the reported metrics are intended to contextualize the present results within the broader literature rather than to support direct, head-to-head performance comparison.

Boundary precision remains crucial for downstream clinical measurements. Inaccuracies in LV or myocardial contours can propagate into derived metrics such as ventricular volumes, myocardial mass, and LVEF, potentially leading to clinically meaningful errors, even when overlap-based metrics remain high [56,57]. The reduction in Hausdorff Distance and mean contour error observed after stacked autoencoder (SAE) refinement indicates improved anatomical plausibility, particularly in basal and apical slices where segmentation errors are most prevalent. The SAE stage is not compensating for weak CNN predictions, but instead serves to enforce shape regularization under weak-contrast cine conditions.

Cine CMR inherently captures temporal cardiac motion [58], yet this study performs segmentation on individual frames without explicit temporal modeling. Temporal consistency is maintained indirectly through shape refinement and contour regularization. While recurrent or transformer-based models could exploit inter-frame dependencies, their exclusion reflects a deliberate design choice prioritizing simplicity, data efficiency, and robustness under limited infarct-positive sample sizes.

Several limitations should be acknowledged. The infarct dataset is small and imbalanced, limiting statistical power and recall performance. RV segmentation, although quantitatively strong, lacks extensive qualitative visualization. Moreover, all experiments were conducted on publicly available datasets, and no external multi-center validation was performed. This lack of multi-center validation may affect generalizability. Future work will therefore focus on expanding infarct-positive cohorts, incorporating multi-center and multi-vendor data, and exploring the integration of complementary CMR sequences (e.g., LGE, T1/T2 mapping) to transition contrast-free MI detection from a methodological framework to a clinically reliable decision-support tool.

## 5. Conclusions

In this study, we developed a deep learning-based framework for the automated segmentation of cardiac structures and MI lesions using contrast-free cine CMR images. The proposed pipeline integrates multiple convolutional CNN architectures with post-processing techniques, addressing the clinical need for rapid, reproducible, and contrast-independent diagnostic tools.

We evaluated shallow, deeper, and larger CNN variants for segmenting the LV cavity, LV myocardium, and RV cavity. All models achieved strong performance, with the best configuration achieving Dice coefficients above 0.90 across most anatomical targets. Additionally, a stacked autoencoder-based post-processing stage was applied to enhance contour accuracy and anatomical consistency. This stage significantly improved the smoothness and clinical plausibility of segmentation outputs. For infarct localization, the Attention U-Net enhanced with SE blocks demonstrated reliable detection from cine CMR alone, achieving an accuracy of 0.98 ± 0.01 and a recall of 0.80 ± 0.02.

These findings address critical limitations in previous studies, such as suboptimal segmentation performance in basal and apical slices, limited generalizability across diverse datasets, and reliance on contrast-enhanced imaging. Furthermore, we systematically investigated the influence of different loss functions and weight initialization strategies, improving training stability and overall segmentation accuracy.

This end-to-end pipeline offers a robust and clinically applicable solution for anatomical and pathological cardiac segmentation using non-contrast CMR. Its adaptability and high performance make it suitable for integration into routine clinical workflows, especially in cases where contrast agents are limited or contraindicated. Future work will focus on multi-sequence data integration, inter-institutional validation, and real-time deployment within clinical environments.

## Figures and Tables

**Table 1:** Composition of the cine CMR datasets used for cardiac structure and myocardial infarction segmentation.

Task	Label Type	Number of Images	Description
LV cavity segmentation	Binary masks	260	Annotated cardiac MRI slices
LV myocardium segmentation	Binary masks	279	Expert-annotated myocardial contours
RV cavity segmentation	Binary masks	266	Annotated right ventricle regions
Myocardial infarction segmentation	Infarction region masks	75	Annotated slices with visible MI pathology

**Table 2:** Architectural comparison of the four CNN-based segmentation models evaluated in this study.

Model Name	Convolutional Layers	Pooling Layers	Fully Connected Layer	Regularization	Output Reshaping	Trainable Parameters
Shallow CNN	Conv2D (100 filters, 11 × 11, ReLU)	AvgPooling2D (6 × 6)	Dense (1024 units, sigmoid)	L2 (λ = 0.0001)	Reshape to (32 × 32)	8,307,624
Max-Pooling CNN	Conv2D (100 filters, 11 × 11, ReLU)	MaxPooling2D (6 × 6)	Dense (1024 units, sigmoid)	L2 (λ = 0.0001)	Reshape to (32 × 32)	8,307,624
Larger CNN	Conv2D (200 filters, 11 × 11, ReLU)	AvgPooling2D (6 × 6)	Dense (1024 units, sigmoid)	L2 (λ = 0.0001)	Reshape to (32 × 32)	16,614,224
Deeper CNN	Conv2D (64 filters, 11 × 11, ReLU) → Conv2D (128 filters, 10 × 10, ReLU)	AvgPooling2D (2 × 2) → AvgPooling2D (2 × 2)	Dense (1024 units, sigmoid)	L2 (λ = 0.0001)	Reshape to (32 × 32)	11,444,992

**Table 3:** Architectural description of attention U-Net.

Block	Layer Type	Output Shape	Kernel Size	Activation	Additional Information
Input	Input Image	128 × 128 × 3	—	—	RGB image
Encoder	Conv2D	128 × 128 × 32	3 × 3	ReLU	Padding: same
	BatchNormalization	128 × 128 × 32	—	—	
	Conv2D	128 × 128 × 32	3 × 3	ReLU	Padding: same
	SE Block	128 × 128 × 32	—	—	Channel-wise feature recalibration
	MaxPooling2D	64 × 64 × 32	2 × 2	—	—
	Conv2D	64 × 64 × 64	3 × 3	ReLU	Padding: same
	BatchNormalization	64 × 64 × 64	—	—	
	Conv2D	64 × 64 × 64	3 × 3	ReLU	Padding: same
	SE Block	64 × 64 × 64	—	—	Channel-wise feature recalibration
	MaxPooling2D	32 × 32 × 64	2 × 2	—	—
Bottleneck	Conv2D	32 × 32 × 128	3 × 3	ReLU	Padding: same
	BatchNormalization	32 × 32 × 128	—	—	
	Conv2D	32 × 32 × 128	3 × 3	ReLU	Padding: same
	SE Block	32 × 32 × 128	—	—	Channel-wise feature recalibration
Decoder	Conv2DTranspose	64 × 64 × 64	2 × 2	—	Upsampling
	Attention Gate	64 × 64 × 64	—	Sigmoid	Feature selection (skip connection)
	Concatenate	64 × 64 × 128	—	—	Skip connection with Encoder features
	Conv2D	64 × 64 × 64	3 × 3	ReLU	Padding: same
	Conv2DTranspose	128 × 128 × 32	2 × 2	—	Upsampling
	Attention Gate	128 × 128 × 32	—	Sigmoid	Feature selection (skip connection)
	Concatenate	128 × 128 × 64	—	—	Skip connection with Encoder features
	Conv2D	128 × 128 × 32	3 × 3	ReLU	Padding: same
Output	Conv2D (final output)	128 × 128 × 1	1 × 1	Sigmoid	Binary segmentation mask

**Table 4:** Pairwise statistical comparison of Dice scores across CNN architectures with Holm–Bonferroni correction (α = 0.05).

Comparison	Mean Dice (Model A)	Mean Dice (Model B)	Nominal *p*-Value	Significant After Holm Correction
Shallow vs. Larger	0.06	0.20	1.08 × 10^−9^	Yes
Shallow vs. Deeper	0.06	0.02	6.02 × 10^−3^	Yes
Shallow vs. Max-pooling	0.06	0.35	3.05 × 10^−2^	No
Shallow vs. ReLU	0.06	0.32	8.95 × 10^−1^	No
Larger vs. Deeper	0.20	0.02	3.05 × 10^−2^	No
Larger vs. Max-pooling	0.20	0.35	1.19 × 10^−1^	No
Larger vs. ReLU	0.20	0.32	5.39 × 10^−9^	Yes

**Table 5:** Loss-function comparison under identical architectural and training settings.

Model (Fixed Architecture)	Loss Function	Dice	IoU
CNN (ROI mask regression)	MSE	0.7740	0.6508
CNN (ROI mask regression)	BCE	0.7672	0.6403
CNN (ROI mask regression)	Dice	0.8094	0.7022
CNN (ROI mask regression)	BCE + Dice	0.8184	0.7104

**Table 6:** Ablation study isolating the contribution of SAE and active contour refinement.

Pipeline Stage	Dice	Hausdorff Distance (HD) (mm)
CNN only	0.66	80
CNN + SAE	0.76	44
CNN + SAE + Active Contour	0.79	26

**Table 7:** Performance metrics (Dice, correlation coefficient, Hausdorff distance) across SAE loss settings.

SAE Loss Setup	Dice Metric (DM)	Hausdorff Distance (HD) (mm)	Correlation Coefficient (CC)
Customized loss + MSE	0.046	90.8 mm	0.25
MSE + MSE	0.345	33.7 mm	0.55
KL divergence + MSE	0.062	38.0 mm	0.35

**Table 9:** Bootstrapped 95% confidence intervals for MI segmentation performance (n = 75 slices).

Metric	Mean	95% CI (Lower–Upper)
Dice	0.77	0.69–0.84
Recall	0.80	0.72–0.87
Precision	0.83	0.76–0.89

**Table 10:** Comparison of segmentation and infarct detection performance across related studies.

Study	Model Used	LV Dice	MYO Dice	RV Dice	Contrast-Free	Infarct Detection
Bai et al. [10] (2018)	Fully Convolutional Network (FCN)	0.94	0.88	0.90	**×**	**×**
Ammar et al. [52] (2021)	Lightweight U-Net	0.92	—	—	**×**	(MLP + RF + SVM ensemble
Penso et al. [25] (2021)	U-Net with Dense Skip Connections	0.944	0.852	0.908	✓	**×**
Chen et al. [55] (2021)	2-stage CNN	—	—	—	**×**	(Scar segmentation)
Zhang et al. [34] (2022)	VNE (GAN + Cine + Native T1)	—	—	—	✓	Acc: 84%, Sen: 77%,
Qi et al. [31] (2024)	Cine-Generated Enhancement (CGE)	—	—	—	✓	(Sen: 91%, Spe: 96%)
Our study (2026)	4 CNN variants + Attention U-Net + SAE	0.93	0.89	0.91	✓	(Acc: 98%, Rec: 80%)

## Data Availability

The datasets generated and/or analyzed during the current study are available in publicly accessible repositories. The Sunnybrook Cardiac Dataset (SCD) is available at https://www.cardiacatlas.org/sunnybrook-cardiac-data/ (accessed on 1 November 2025), and the Kaggle Second Annual Data Science Bowl dataset is available at https://www.kaggle.com/datasets/adarshsng/heart-mri-image-dataset-left-atrial-segmentation/data (accessed on 1 November 2025).
